# The effect of galvanization and potassium iodide iontophoresis of the throat and larynx on thyroid parameters: a randomized controlled trial

**DOI:** 10.1038/s41598-021-95145-w

**Published:** 2021-08-02

**Authors:** Jolanta Zwolińska, Barbara Augustyn, Katarzyna Baj, Jadwiga Krukowska

**Affiliations:** 1 Institute of Health Sciences, Medical College, University of Rzeszow, Rzeszow, Poland; 2 Scientific Club of Physical Energy Used in Physiotherapy, University of Rzeszow, Rzeszow, Poland; 3 St. Hedvig Clinical Provincial Hospital No. 2, Rzeszow, Poland

**Keywords:** Endocrinology, Medical research

## Abstract

Few studies have assessed the application and side effects of potassium iodide (KI) iontophoresis. Using a double-blinded randomized controlled trial with a 1:1 parallel-group, we investigated the effect of galvanization and the KI iontophoresis in the throat and larynx on three thyroid parameters. A total of 50 healthy volunteers with normal TSH, FT_3_, and FT_4_ levels and lacking focal changes in the thyroid ultrasonography were subjected to 10 electrotherapy treatments. The TSH, FT_3_, and FT_4_ levels were determined prior to the 10 electrotherapeutic treatments (T1), 2-weeks after treatment (T2) and 6-months after treatment (T3). At T2 and T3, both groups had normal levels of TSH, FT_3_, and FT_4_. Regarding the change of TSH, FT_3_, and FT_4_ levels between T1 vs. T2 and T1 vs. T3, no significant differences between the galvanization and iontophoresis groups were found. However, both groups had lower levels of all three hormones at T3. Together, these data indicate that KI iontophoresis does not affect thyroid hormone levels in the short- nor long-term. Additional follow-up studies with larger groups are required to better confirm the safety of galvanization and iontophoresis procedures in the pharynx and larynx.

**Trial registration** ClinicalTrials.gov (NCT04013308; URL: www.clinicaltrials.gov). Day of first registration 09/07/2019.

## Introduction

Iodine is essential for the production of the thyroid hormones, triiodothyronine (T_3_) and thyroxine (T_4_), which are essential for systemic homeostasis. Furthermore, these hormones play an important role in the metabolism, growth and maturation of various organs and systems, especially the nervous system^1,2^. According to the recommendations of the World Health Organization (WHO) and the United States Institute of Medicine (IOM), the recommended daily intake (RDI) of iodine for adults is approximately 150 μg^2–4^. Additionally, both low and high iodine intake is associated with an increased risk of thyroid disease, and an optimal daily intake of iodine is important to prevent thyroid diseases^3,5,6^. In order to assess thyroid functioning, the level of thyroid-stimulating hormone (TSH) secreted by the pituitary gland, free triiodothyronine (FT_3_) and free thyroxine (FT_4_) are routinely measured in the blood^7–10^.

Medical reference books enumerate various applications for the appropriate use of physiotherapeutic procedures for throat and larynx conditions^11–14^. However, these recommendations are not appropriately grounded in published scientific data. The most common throat and larynx conditions in which physical therapy is prescribed include the paralysis of the vocal cords of the larynx, chronic pharyngitis and laryngitis, dysphonia, and the treatment of vocal cord nodules. Patients are most often referred for physical therapy treatments for pharynx and larynx issues by medical otolaryngologic and phoniatric specialists^15^.

Potassium iodide (KI) iontophoresis is used in the therapy of throat and larynx conditions, arthritis, arthrosis, scar contractures, and hypertrophic scars^15–18^. Further, the application of KI iontophoresis facilitates a reduction in antibiotic therapy, which is of great importance to prevent excessive antibiotic use^19–21^. Further, few studies have investigated the possible side effects of KI iontophoresis in the pharynx and larynx locality.

This study aimed to assess the impact of galvanization and KI iontophoresis procedures performed on the pharynx and larynx as well as the level of TSH, FT_3_ and FT_4_ hormones.

## Results

### Flow of participants

Group G included 19 women and 7 men, while group I included 20 women and 4 men.

At baseline, the randomization process yielded no major differences between the groups (G and I) regarding gender (*p* = 0.3818) (data not shown), body mass index, and age (Table 1).

**Table 1:** Characteristics of the studied groups in terms of body mass index and age.

Analyzed feature	Type of therapy				*p-*value
	Galvanization		Iontophoresis		
	$\overline{x} \pm s$	min–max	$\overline{x} \pm s$	Min–max	
BMI	22.2 ± 2.8	17.9–27.9	21.5 ± 2.1	18.4–26.6	**0.2724**
Age[years]	21.8 ± 1.0	20.0–25.0	22.0 ± 1.0	21.0–24.0	**0.6234**

*p*-test probability value calculated using the independent samples t-test.

### Short-term effects of electrotherapy (n = 50)

One person from group G demonstrated a significant decrease in TSH level (0.101 µIU/mL) at T2 and a significant increase in this value (11.6 µIU/mL) at T3. This person was excluded from further analysis. The TSH, FT_3_ and FT_4_ levels were all within the normal range for the remaining participants. There were no significant differences between groups G and I in TSH level at T1 and T2. Further, the changes in TSH levels recorded from T1 to T2 were similar (Table 2).

**Table 2:** Changes in TSH, FT_3_ and FT_4_ levels in the study groups from T1 to T2.

Thyroid parameters	Type of therapy						*p-*value
	Galvanization			Iontophoresis			
	Mean (95% C.I.)	Me	*s*	Mean (95% C.I.)	Me	*s*	
**TSH [µIU/mL]**							
Before electrotherapy	2.26 (1.84; 2.68)	2.03	1.01	2.28 (1.99; 2.57)	2.22	0.71	0.9493
After electrotherapy	1.96 (1.59; 2.32)	1.88	0.89	1.93 (1.61; 2.24)	1.75	0.77	0.8968
Therapy effect	− 0.31 (0.61; 0.00)	− 0.22	0.74	− 0.35 (− 0.66; − 0.04)	− 0.40	0.75	0.8278
**FT**_**3**_** [pg/mL]**							
Before electrotherapy	3.43 (3.28; 3.59)	3.42	0.37	3.23 (3.03; 3.42)	3.36	0.47	0.0910
After electrotherapy	3.42 (3.20; 3.63)	3.38	0.52	3.37 (3.13; 3.60)	3.34	0.57	0.7611
Therapy effect	− 0.02 (− 0.18; 0.14)	− 0.06	0.39	0.14 (− 0.06; 0.34)	0.11	0.48	0.2051
**FT**_**4**_** [ng/dL]**							
Before electrotherapy	1.31 (1.25; 1.38)	1.31	0.16	1.29 (1.24; 1.33)	1.26	0.12	0.5124
After electrotherapy	1.31 (1.22; 1.39)	1.25	0.21	1.26 (1.21; 1.32)	1.29	0.14	0.3822
Therapy effect	− 0.01 (− 0.07; 0.06)	-0.03	0.16	− 0.02 (− 0.07; 0.02)	0.00	0.11	0.6473

*p*-test probability value calculated using the independent samples t-test.

A decrease in the TSH levels (group G: p = 0.0513, group I: p = 0.0281) was observed in the individual groups (Table 2).

The FT_3_ level before electrotherapy was slightly higher in group G. However, there were no significant differences in the FT_3_ level between groups G and I from T1 to T2 (Table 2). No significant changes were found in the FT_3_ level within the individual groups (group G: p = 0.8311, group I: p = 0.1521). Additionally, no significant differences in the levels of FT_4_ were observed between groups G and I from T1 to T2 (Table 2). Further, no significant changes were found in the FT_4_ levels within the studied groups from T1 to T2 (group G: p = 0.8710, group I: p = 0.3209).

The increase or decrease in thyroid hormones was analyzed in individual cases in both groups (G and I) (Table 3).

**Table 3:** The number and percentage of people for whom the given parameter increased and decreased from T1 to T2.

Grouped features	Type of therapy								*p-*value
	Galvanization				Iontophoresis				
	Decrease		Increase		Decrease		Increase		
	*N*	%	*N*	%	*N*	%	*N*	%	
TSH [µIU/mL] (change)	15	60	10	40	18	72	7	28	0.3705
FT_3_ [pg/mL] (change)	14	56	11	44	10	40	15	60	0.2575
FT_4_ [ng/dL] (change)	14	56	11	44	12	48	13	52	0.5713

*p*-test probability value calculated using the chi-square test of independence.

TSH decreased in 60% and 72% of participants, in group I and G, respectively. However, there were no significant differences in electrotherapeutic effects between group G and group I.

### Long-term effects of electrotherapy (n = 36)

In both groups, the TSH levels at T3 were within the normal range. At all three time points (T1, T2 and T3), the TSH levels, as well as changes in those levels, were similar groups G and I in the periods: T1 vs. T2, T2 vs. T3, and T1 vs. T3 (Table 4).

**Table 4:** Changes in TSH, FT_3_ and FT_4_ levels in the study groups at T1 vs. T2, T2 vs. T3, and T1 vs. T3.

Thyroid parameters	Type of therapy						*p-*value
	Galvanization (*n* = 21)			Iontophoresis (*n* = 15)			
	Mean (95% C.I.)	Me	*s*	Mean (95% C.I.)	Me	*s*	
**TSH [µIU/mL]**							
Before electrotherapy (T1)	2.18 (1.72; 2.64)	1.79	1.01	2.42 (1.99; 2,86)	2.31	0.79	0.4515
After electrotherapy (T2)	2.01 (1.65; 2.37)	1.91	0.79	1.95 (1.57; 2.33)	1.91	0.68	0.8238
6 months later (T3)	1.64 (1.36; 1.92)	1.88	0.61	1.91 (1.46; 2.37)	1.79	0.82	0.2640
(T1–T2)	− 0.17 (− 0.52; 0.18)	0.01	0.76	− 0.47 (− 0.94; 0.00)	− 0.54	0.85	0.2819
(T2–T3)	− 0.37 (− 0.61; − 0.12)	− 0.33	0.54	− 0.04 (− 0.59; 0.51)	− 0.33	0.99	0.2108
(T1–T3)	− 0.54 (− 0.97; − 0.12)	− 0.39	0.93	− 0.51 (− 1.02; 0.00)	− 0.31	0.92	0.9185
**FT**_**3**_** [pg/mL]**							
Before electrotherapy (T1)	3.42 (3.25; 3.59)	3.42	0.37	3.29 (3.08; 3.51)	3.36	0.39	0.3306
After electrotherapy (T2)	3.43 (3.19; 3.67)	3.38	0.53	3.40 (3.08; 3.72)	3.37	0.57	0.8675
6-months later (T3)	3.14 (2.95; 3.33)	3.14	0.42	3.00 (2.80; 3.19)	3.03	0.35	0.2865
(T1–T2)	0.01 (− 0.17; 0.20)	− 0.03	0.40	0.11 (− 0.15; 0.37)	0.01	0.47	0.5220
(T2–T3)	− 0.29 (− 0.56; − 0.03)	− 0.23	0.58	− 0.40 (− 0.69; − 0.12)	− 0.27	0.52	0.5562
(T1–T3)	− 0.28 (− 0.45; − 0.11)	− 0.27	0.37	− 0.30 (− 0.50; − 0.09)	− 0.14	0.37	0.8891
**FT**_**4**_** [ng/dL]**							
Before electrotherapy (T1)	3.42 (3.25; 3.59)	3.42	0.37	3.29 (3.08; 3.51)	3.36	0.39	0.3306
After electrotherapy (T2)	3.43 (3.19; 3.67)	3.38	0.53	3.40 (3.08; 3.72)	3.37	0.57	0.8675
6-months later (T3)	3.14 (2.95; 3.33)	3.14	0.42	3.00 (2.80; 3.19)	3.03	0.35	0.2865
(T1–T2)	0.01 (− 0.17; 0.20)	− 0.03	0.40	0.11 (− 0.15; 0.37)	0.01	0.47	0.5220
(T2–T3)	− 0.29 (− 0.56; − 0.03)	− 0.23	0.58	− 0.40 (− 0.69; − 0.12)	− 0.27	0.52	0.5562
(T1–T3)	− 0.28 (− 0.45; − 0.11)	− 0.27	0.37	− 0.30 (− 0.50; − 0.09)	− 0.14	0.37	0.8891

*p*-test probability value calculated using the independent samples *t*-test.

At T3, a significant decrease in TSH levels was observed in both groups (G and I) compared to the two previous time points (T1 and T2). From T1 to T3 there was a significant increase of the TSH levels in group G (p = 0.0151), while in group I, there was a convincing decrease in TSH levels, however, this observation was not significant (p = 0.0502).

Similar changes were recorded when assessing the FT_3_ and FT_4_ levels. Namely, there were no significant differences in the FT_3_ levels between groups G and I (Table 4). However, in both groups, there were significant differences in the FT_3_ levels at T3 as well as the prior two timepoints. This was indicative by the significant decrease in the FT_3_ levels from T2 to T3 (group G: p = 0.0325, group I: p = 0.0095) and from T1 to T3 (group G: p = 0.0024, group I: p = 0.0074). Additionally, there were no significant differences in the FT_4_ levels observed between groups G and I, respectively (Table 4). From T1 to T3, there was a significant decrease in the FT_4_ levels in group G (p = 0.0123) and group I (p = 0.0016).

## Discussion

Few studies have elaborated on the application and possible side effects of galvanization or KI iontophoresis directed at the pharynx and larynx.

This study aimed to evaluate the effect of galvanization and transdermal iodine application on the levels of TSH, FT_3_, and FT_4_. To achieve this, the amount of iodine that penetrated the tissues during iontophoresis was calculated based on Faraday’s first law of electrolysis with the assumption that the entire current consists exclusively of the ions of the drug substance^22^. If numerous competing ions, including parasitic ones, are present at the site of administration, the amount of the basic drug substance penetrating the tissues is reduced.

Puttemans et al.^23^ confirmed the penetration of KI into tissue using galvanic current. In that study, the authors estimated that approximately 10% of the KI used for iontophoresis penetrated deep into the tissues during transdermal administration. After a series of 10 iontophoresis treatments with KI, the mean concentration of iodine in the thyroid gland was observed to increase by approximately 30%^23^.

The acceptable upper level of the daily iodine supply is 1100 µg; however, a higher intake is well tolerated^2^. Further, the ingestion of KI protects against irradiation of the thyroid gland after exposure to radioactive iodine^24,25^. Verger et al.^24^ reported that daily consumption of 15 mg KI provided > 90% protection to the thyroid gland^24^. Furthermore, Zanzonico and Becker^25^ reported that the blockage of the thyroid gland by oral KI at a dose of 50–100 mg may effectively reduce thyroid radiation^25^. This was further substantiated in the study by Bacher et al.^26^ who showed that a daily intake of 100 mg of KI was able to prevent the radiation of the thyroid gland^26^.

In the present study, 200 mg of KI was used for a single iontophoresis treatment. Using Faraday’s law I of electrolysis, we estimated that during one iontophoresis treatment, less than 6.1927461 mg of KI was introduced into the tissues. Further, this calculation assumed that it is impossible to eliminate all competing ions in the electric field created between the treatment electrodes. Together, the data from this study indicate that an iodine dose used during iontophoresis had no additional effect on the levels of TSH, FT_3_ and FT_4_ hormones.

In the current study, the proportion of participants with decreased TSH, FT_3_ and FT_4_ levels was similar in both groups, which further substantiates the conclusion that KI iontophoresis does not induce significant changes in the production of these important hormones.

Further, previous studies indicate that differing quantities of diverse iodine sources can affect thyroid assessment. For example, the application of high amounts of iodine in x-ray contrast media can induce changes in thyroid function parameters^27–29^. Other studies which have investigated the effects of iodine nutrition on the thyroid have highlighted the need for the ongoing monitoring of iodized salt and other dietary iodine sources; a strategy that should be implemented to prevent excess as well as insufficient iodine nutrition^30,31^. The participants of our study were completely healthy. Furthermore, they were not subjected to any medical procedures which may have exposed them to high doses of iodine. Additionally, all participants did not imbibe excessive iodine through their nutrition.

During iontophoresis, the thyroid gland is influenced by direct, galvanic current. The specific biological effect of electric fields on tissues is still largely unknown. However, the flow of current can affect tissues and organs^32^. Additionally, living cells can be induced to migrate by applying a small dose of direct current (galvanotaxis)^33^. The human body, as a bioelectrical circuit, is characteristic of an anisotropic conductor. During the flow of current, thermal energy is released and the affected tissues become hyperemic^18,34^. According to Joule-Lenz’s law, the amount of heat released is directly proportional to the square of the current intensity, its flow time and tissue resistance^34^. Previous studies have shown that an electric current can cause the depolarization of the cell membrane of excitable cells^32^. Additionally, it also influences the pH of the tissues. The electrochemical changes around the negative electrode (an alkaline environment produced by OH^-^ ions) are more severe compared to the changes around the positive electrode (an acidic environment produced by H^+^ ions)^35^. In our study, the heat generated, and electrochemical changes induced, were minor and did not pose a threat to the study participants. We used a low current of 2 mA during the procedure, which corresponds to the perception threshold for direct current. This value is reported to be approximately 1.5 mA for women and 2.5 mA for men^32,34^.

Gierlotka^34^ emphasized that direct current flowing through tissues for a specific duration can cause pathological changes even if its intensity does not exceed the perception threshold^34^. Additionally, in a recent study, Dechent et al.^32^ demonstrated that the negative effects of current flow through tissues may be immediately apparent but can be delayed by several months, or even years^32^. The reduction in the values of all three hormones assessed in this study, particularly those data acquired at T3, suggests that the assessment of possible side effects of electrotherapeutic treatments in the throat and larynx should be the subject of future studies in physiotherapy involving larger groups of subjects.

This study deviated slightly from the adopted research protocol in that in group G, one patient received a lower current (1.5 mA) due to the intense feeling of current vibrations experienced. Additionally, on the penultimate day of treatment, one patient from group G underwent two galvanization treatments (in the morning and evening). Lastly, one person from group G had a second blood test one week later (3-weeks after electrotherapy).

## Conclusion

In summary, potassium iodide iontophoresis treatments had no additional effect to the galvanic current on the levels of TSH, FT_3_ and FT_4_ in the long term.

Additionally, the high frequency of focal lesions in the thyroid gland observed in the study group via ultrasonography highlights the need for routine thyroid examinations in patients referred for either galvanization or iontophoresis in the pharynx and larynx area. Further, the evaluation of possible side effects of these treatments when used on the pharynx and larynx requires additional and future follow-up studies. These studies should ideally include larger groups of subjects.

## Material and methods

### Design

All data from this study were acquired through a double-blinded, 1:1 parallel-group, randomized controlled trial. Agreeable participants that had no contraindications to electrotherapy in the neck area were selected for, or excluded from, participation in the study through a computer-generated randomization list. Qualified participants were then subjected to ultrasonography of the thyroid gland and had several hormone levels (TSH, FT_3_ and FT_4_) determined. Thereafter, two study groups were assigned to two different interventions (galvanization—group G and iontophoresis—group I). These were formed by subsequent randomization. The participants of the study were not informed as to which group they were assigned to.

### Participants

This study was conducted in the Centre for Innovative Research in Medical and Natural Sciences, University of Rzeszow and the members of the Scientific Circle of Physical Energy Used in Physiotherapy were therapists.

Students in their third, fourth and fifth year of physiotherapy were invited to participate in the study. Written information was provided which detailed the purpose and course of the study. Further, the choice for the participants to withdraw from the study was emphasized at every stage.

The inclusion criteria were:

- Informed written consent of the patient to participate in the study.
- No contraindications to electrotherapy in the throat and larynx.
- Normal results of ultrasonography of the thyroid and hormone levels (TSH, FT_3_, and FT_4_).
- No neoplastic and thyroid diseases in the study participant or their immediate family.

The exclusion criteria were:

- Poor tolerance of electrotherapy treatments.
- Breaks between consecutive treatments longer than 3-days.
- Failure to complete a series of iontophoresis/galvanization treatments.
- The use of any stimulants during the observation period.

### Intervention

Participants were subjected to a series of 10 electrotherapy treatments (galvanization or iontophoresis) according to the result of randomization. For the cathodic galvanization treatment, distilled water (placebo) was used for the treatment with a current of 2 mA for 30 min. For the cathodic iontophoresis treatment, 10 mL of 2% KI solution (200 mg of KI) was used with a current 2 mA for 30 min. After each treatment, the condition of the skin in the treatment area was assessed to exclude any potential symptoms of an iodine allergy.

During the iontophoresis procedure, 6.1927461 mg of potassium iodide, including 4.73409324 mg of iodine was introduced into the tissues (Supplementary Appendix A). For electrotherapy, a 4 cm × 5 cm active electrode placed at the throat area (current density was 0.1), and a 5 cm × 6 cm passive electrode was placed at the nape. The current density was 0.1 mA/cm^2^ and 0.66 mA/cm^2^ for the throat and nape pads, respectively.

### Outcome measures

Before the study, all participants had an ultrasonography examination of the thyroid gland performed by a radiologist.

**Figure 1:**
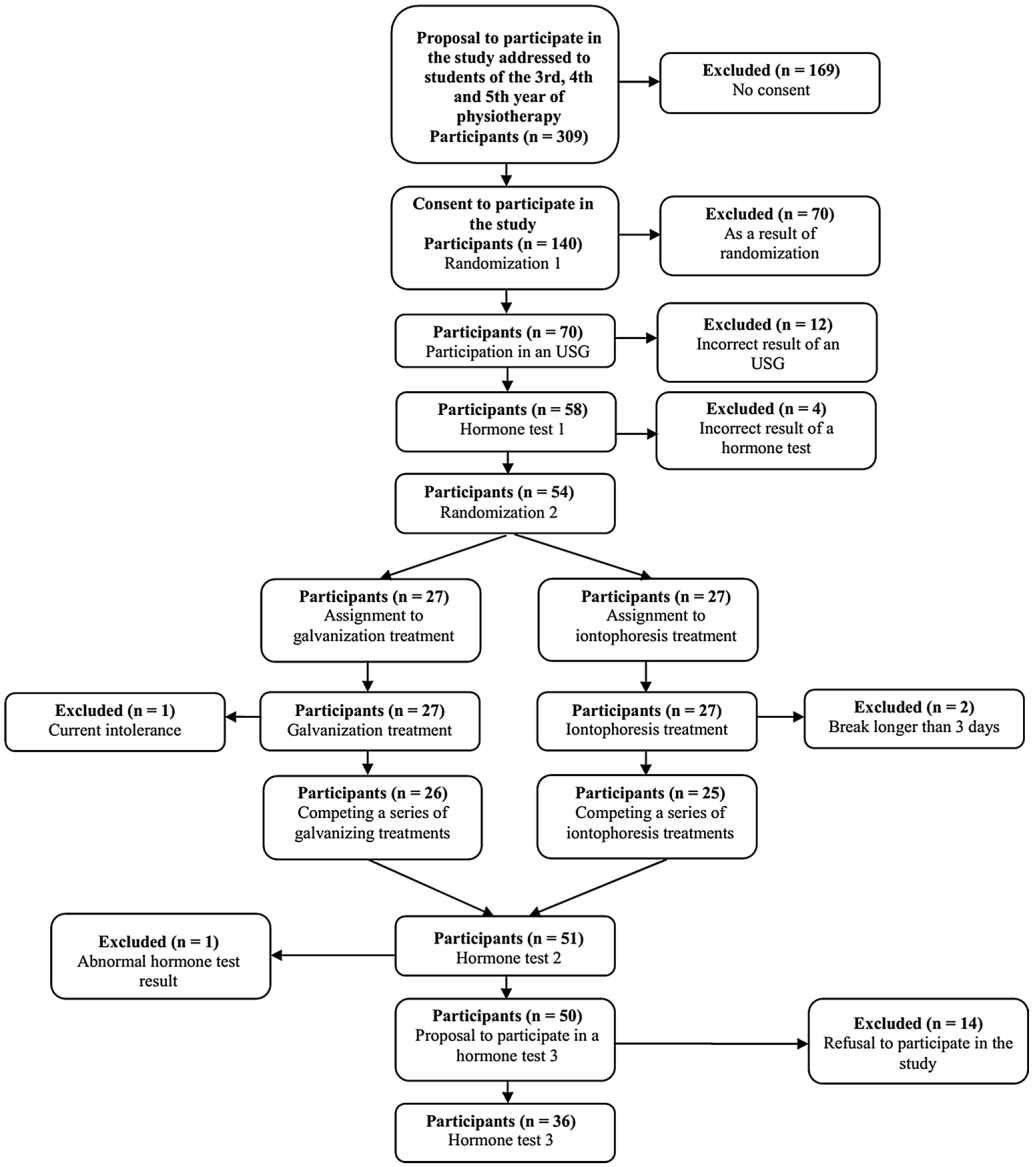
Design and flow of participants in the study.

The outcomes assessed were the levels of TSH, FT_3_, FT_4_ hormones (Supplementary Appendix B). Hormone levels were tested before 10 electrotherapy treatments (T1), 2 weeks after electrotherapy (T2), and 6 months after electrotherapy (T3) (Fig. 1). All hormone tests were performed in the same laboratory.

### Data analysis

Initially, the data obtained from the 50 individuals who participated from T1 to T2 were analyzed. The level of TSH, FT_3_ and FT_4_ was compared before electrotherapy (T1), after electrotherapy (T2), as well as the effect of electrotherapy between group G (galvanization) and group I (iontophoresis). The statistical significance of the electrotherapy effect was also assessed within each group (G and I).

Next, the data obtained from 36 participants who took part in three subsequent tests were analyzed (T1 to T3). The significance of changes in individual parameters was assessed in the period between the first and second tests (T1 to T2), between the second and third tests (T2 to T3), and between the first and third tests (T1 and T3) (electrotherapy effects) as well as separately for each group (G and I). The level of individual parameters was compared between groups I and G for each study, as well as for the observed changes (effects of different electrotherapy treatments).

As the distributions of the studied values did not differ significantly from normality, the independent samples t-test was used to evaluate differences between groups and the paired sample t-test was uses to assess the significance of the parameters within groups. Additionally, the chi-square test of independence was used to assess the varying frequency of decreases or increases in the values of individual parameters in both groups.

The sample size was determined for TSH. Based on the preliminary examination for 10 people, the mean TSH value was determined at the level of 2.6 µIU/mL with the standard deviation amounting to about 1.2 µIU/mL. It was assumed that the sample size should detect a change between tests at 50% of the variation in the first test (i.e., 0.6 µIU/mL) at a significance level of 0.05 and 80% of test power. Given these parameters, a minimum sample size of 21 was calculated. To account for any aberrations, groups of 25 participants were included in the analysis.

The analysis, interpretation of results and statistical significance was determined at p ≤ 0.05 (*p < 0.05 **p < 0.01; ***p < 0.001) using *STATISTICA* software.

### Ethics approval

The study was conducted in accordance with the Declaration of Helsinki, and the protocol was approved by the Ethics Committee of University of Rzeszow (resolution No. 2018/05/04). Participants gave written informed consent before fata collection began.

## Supplementary Information

Supplementary Information.
