# Supplementary material for: The effect of galvanization and potassium iodide iontophoresis of the throat and larynx on thyroid parameters: a randomized controlled trial
Source: Sci Rep. 2021 Aug 2;11:15590. doi: 10.1038/s41598-021-95145-w (PMC8329291; doi:10.1038/s41598-021-95145-w)
Supplement: Supplementary file 1 — Supplementary Information. [file 41598_2021_95145_MOESM1_ESM.docx]

Appendix A. Faraday's first law of electrolysis

| m = k . i . t | Faraday's first law of electrolysis |
| --- | --- |
| m | mass of substance evolved on the electrode |
| i | the amperage of current flowing in the circuit |
| t | current flow time |
| k = R/F | electrochemical equivalent [k_KI =_ 0,00172020725] |
| R | chemical equivalent, i.e. the mass of 1 mole [Mmol_KI =_ 166g/mol] |
| F | The Faraday constant [F = 96 500 C] |

Appendix B. Examined hormones

| TSH [µIU/ml] | micro international unit /mililiter |
| --- | --- |
| FT3 [pg/ml] | picogram/mililiter |
| FT4 [pg/ml] | picogram/mililiter |
